# Paraplegia After Seat Belt Abdominal Aorta Occlusion Associated With L4 Chance Fracture

**DOI:** 10.7759/cureus.66860

**Published:** 2024-08-14

**Authors:** Orlando De Jesus, Heidy L Garcia-Irizarry, Edna M Castañeda-Torres

**Affiliations:** 1 Neurosurgery, University of Puerto Rico, Medical Sciences Campus, San Juan, PRI

**Keywords:** occlusion, blunt trauma, seat belt, chance fracture, aorta, paraplegia

## Abstract

Blunt traumatic injury to the chest or abdomen can produce injury to the aorta, which can compromise perfusion to the lower spinal cord. This report presents the case of a seat belt-restrained driver who sustained blunt abdominal trauma and progressive paraplegia. The trauma produced an acute occlusion of the abdominal aorta associated with an L4 Chance fracture and multiple bowel injuries. The Chance fracture occurred two levels below the aortic occlusion. The significant aortic atherosclerosis changes in this patient could have triggered the complete occlusion two levels above the fracture. An aortic injury associated with a vertebral fracture represents a severe and potentially lethal condition. Lower limb ischemia in the setting of a blunt abdominal injury could lead to a high diagnostic suspicion of abdominal aortic injury. Treatment of the vascular injury should be performed without delay to prevent or reduce permanent neurological deficits and ischemic injuries.

## Introduction

Blunt traumatic injury to the chest or abdomen can produce injury to the aorta, which can compromise perfusion to the lower spinal cord. With blunt trauma, injury to the thoracic aorta is far more frequent than injury to the abdominal aorta [1,2]. Injury to the abdominal aorta after blunt traumatic injury occurs in approximately 0.01%-0.07% of the patients [3-5]. Distal spinal cord perfusion is usually dependent on a few major radicular arteries, with the great radicular artery of Adamkiewicz being the most important, which originates from an intercostal artery between T8 and L1 in 89% of individuals [6]. Acute occlusion of the aorta at the lower thoracic segment or the abdominal aorta is likely to involve the Adamkiewicz artery, causing irreversible lower spinal cord ischemia if not treated promptly. Paraplegia after blunt traumatic aortic injury occurs in approximately 2% of the patients [7,8]. However, in these patients, aortic surgical repair can produce paraplegia in 9.9% of the cases [7]. This report presents the case of a seat belt-restrained driver who sustained blunt abdominal trauma and progressive paraplegia. The trauma produced an acute occlusion of the abdominal aorta associated with an L4 Chance fracture and multiple bowel injuries.

## Case presentation

A 51-year-old male driver, who was wearing a lap and shoulder seat belt, was brought from the scene after a motor vehicle accident when he crashed into a fixed object. He had a history of hypertension and type 2 diabetes mellitus. On admission to the trauma emergency room, he was alert and oriented but unable to move both his lower extremities. Initial examination of the abdomen showed visible bruises and a large contusion along the inferior aspect of the left abdominal wall, which was compatible with a seat belt injury. The abdomen was distended, tympanic, and tender to palpation. Lower extremity pulses were palpable but noticeably decreased 1+ bilaterally at the femoral, popliteal, and dorsalis pedis arteries. His legs had adequate color and were warm to palpation. Motor strength was absent (0/5) in both lower extremities. Knee and ankle deep tendon reflexes were absent bilaterally. Rectal tone was absent; however, there was no saddle anesthesia. A deep pain sensation was present in the left leg but was diminished in the right below the inguinal region without a specific sensory level. Proprioception was absent in the legs.

**Figure 1:**
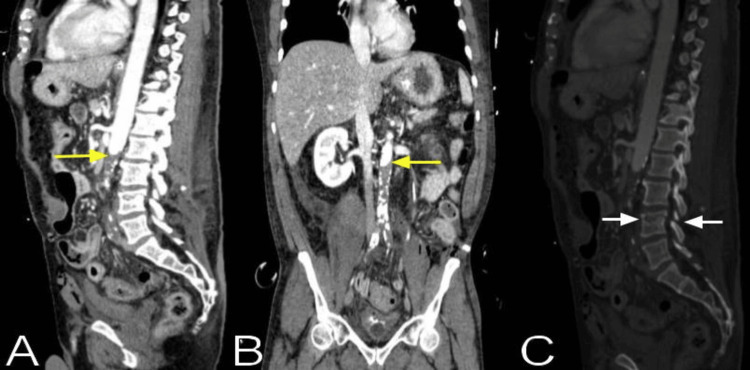
The abdominal CT angiography sagittal (A) and coronal (B) views show an abrupt cutoff of the infrarenal aorta (yellow arrows) with an occluding thrombus immediately below the take-off of the left renal artery at the level of the L2-L3 vertebral junction without distal reconstitution, with significant aortic atherosclerosis changes below the occlusion. The CT bone reconstruction, sagittal (C) view, demonstrates an L4 superior vertebral body fracture, with the fracture line extending throughout the superior endplates and bilateral pedicles (white arrows).

An emergency abdominal sonographic study revealed free fluid in the Morison's pouch, splenorenal fossa, and perivesical areas. Abdominal CT angiography showed an abrupt cutoff of the infrarenal aorta with an occluding thrombus immediately below the take-off of the left renal artery at the level of the L2-L3 vertebral junction with complete occlusion of bilateral common, external, and internal iliac arteries without distal reconstitution (Figure 1). The study also demonstrated an L4 superior vertebral body fracture, with the fracture line extending throughout the superior endplates, bilateral pedicles, and articular processes, compatible with a Chance fracture without retropulsion or angulation. As the pulses were palpable, although decreased, the surgeons thought that the abdominal occlusion was chronic and decided to explore the abdomen and repair the bowel injuries. Due to the patient's unstable and critical condition, a spine MRI was not performed.

An emergency exploratory laparotomy was performed, revealing a left lower quadrant abdominal wall hernia with complete transection of the left rectus abdominis muscle and underlying tissues, with a proximal ileum bucket-handle injury eviscerating through the hernia and presenting a severe ischemic injury. Approximately 15 cm of the proximal ileum was resected. A complete transection of the sigmoid colon was encountered, and proximal and distal partial colectomies were performed. Given the critical condition involving acidosis, hypothermia, and coagulopathy, a temporary abdominal closure was done to have a second look and perform a colostomy upon optimization.

On the operation the following day, a small segment of the sigmoid colon was found to be ischemic and was resected. The proximal segment of the sigmoid colon was exteriorized through the left rectus abdominis defect, and a colostomy was performed in a modified Brooke fashion. A Hartmann pouch was performed at the distal segment. Following the second surgery, mottling was noted on the lateral aspect of each lower extremity and both feet. Both legs were cold and pulseless. Motor function and sensation were absent in the lower extremities. As more than 72 hours had elapsed since the trauma, revascularization was deemed useless. The patient's postoperative course was complicated by renal failure attributed to myoglobinuria and severe hyperkalemia. Due to the poor overall prognosis, the patient's family opted for only supportive and palliative care. He expired three days later.

## Discussion

The overall mortality rate of blunt abdominal aortic artery injury has been reported to be between 15% and 41% [1,2,9-14]. Mortality rates vary depending on the severity of the associated injuries, which may include mesenteric, bowel injuries, and thoracolumbar fractures. The aortic injury spectrum can range from unstable full-thickness laceration, symptomatic stable intimal dissection with occlusion, asymptomatic full-thickness laceration, or intimal dissection without occlusion [2-4]. Blunt abdominal aortic disruption is frequently seen concurrently with a thoracolumbar spine fracture [4]. In our patient, the aortic occlusion occurred at the level of the L2-L3 vertebral junction; however, the Chance fracture was at the L4 vertebra. Chance fractures were first described in 1948 [15]. Since then, Chance fractures have been more recognized, secondary to the routine use of seat belts [1,2,16]. Chance fractures are characterized by the failure of the entire spinal column that could be associated with vertebral dislocation and displacement, causing stretches and strains of the aorta [2,13]. However, blunt abdominal trauma with an aortic injury associated with Chance fractures is an uncommon presentation [1,2,9,10,17,18]. With a blunt aortic injury, spinal fractures presenting distraction, displacement, and translation of the bony elements are more common [4,13,19].

The "seat belt syndrome" was first described by Garrett and Braunstein in 1962 to describe intraabdominal visceral injuries and fractures of the lumbar spine related to automobile seat belts [20]. Using a lap and shoulder seat belt, which provides a three-point fixation, does not eliminate the odds of sustaining a Chance fracture [1]. Thoracolumbar fractures frequently involve a distractive mechanism, with both distractive and translational components [4,13]. The abdominal aorta is protected from injury by the rigid vertebral column and its deep retroperitoneal position; however, with spine fractures, the aorta is vulnerable to mechanical forces transmitted to it [4].

Aortic injury can occur after a rapid deceleration resulting in longitudinal stretching of the aorta with an intimal tear or when the aorta is compressed against the spinal column when the fractured spine is distracted and hyperflexed [9,10,13]. Thoracolumbar spine fractures with aortic injury most commonly occur at the 11th thoracic to the second lumbar vertebrae [1,13]. The presence of aortic atherosclerosis, which weakens the intima, may increase the susceptibility to aortic injury after blunt abdominal trauma [1,2]. In our patient, the Chance fracture occurred at the L4 level, two levels below the aortic occlusion. The significant aortic atherosclerosis changes could have triggered the complete occlusion above the fracture. The patient presented with notable neurological deficits upon initial examination; however, complete motor and sensory deficits and lower limb ischemia occurred two days after the trauma. The Chance fracture did not cause the initial neurological deficits, as its L4 location would have only produced motor deficits distally in the legs. In Gouveia et al.'s review of thoracolumbar fractures with blunt traumatic aortic injuries in adult patients, 36% had neurological disorders, with 25% presenting a complete motor and sensory deficit [13]. Lower limb ischemia could occur in up to 60% of the patients with injury to the abdominal aorta after blunt traumatic injury [14].

CT angiography of the abdomen usually identifies most aortic injuries. However, sometimes, the aortic injury could be identified during emergency exploratory laparotomy. Vascular repair of the aorta should be the priority in treatment to ensure hemodynamic stabilization of the acute patient and correct the associated limb ischemia [13,14,17]. Repair of the aortic injury within six hours is recommended as this time frame confers the best possibility for limb salvage and full neurologic recovery [12]. Spinal fixation is not recommended before the vascular repair [13]. Although neurological recovery is poor in most cases, reduction and stabilization of the spinal injury should be performed at the earliest possible time to allow safe mobilization of the patient and avoid additional injury to the aorta and spinal cord [19].

Vascular findings can be overshadowed by neurologic deficits and abdominal visceral injuries [2]. In cases with aortic injuries, neurological deficits can be incorrectly attributed to the spinal fracture, delaying diagnosis of the aortic injury [2]. In patients with a Chance fracture, the diagnosis of aortic injury is frequently overlooked or delayed [18]. In our patient, the acute aortic injury was not initially recognized due to the severity of the abdominal and neurologic injuries. A high suspicion for an aortic injury should be considered, especially in patients with diminished distal pulses, in whom numbness, decreased pinprick sensation, or motor weakness appear following the injury.

## Conclusions

An aortic injury associated with a vertebral fracture represents a severe and potentially lethal condition. A patient who sustains seat belt Chance fractures presenting neurological deficits should be thoroughly studied for associated aortic injuries. Lower limb ischemia in the setting of a blunt abdominal injury could lead to a high diagnostic suspicion of abdominal aortic injury. Diagnostic studies and treatment of the aortic injury should be performed without delay to prevent or reduce permanent neurological deficits and ischemic injuries.
